# Supplementary material for: Assessment of the influential mechanisms of adolescent risk behaviors and protective and risk factors among high school students in Finnmark, Arctic Norway
Source: PLoS One. 2025 Jul 10;20(7):e0328066. doi: 10.1371/journal.pone.0328066 (PMC12244561; doi:10.1371/journal.pone.0328066)
Supplement: Attachment — Submitted filename: Response letter to reviewer.docx [file pone.0328066.s002.docx]

**Response to the Reviewers’ Comments and Concerns**

**Comments on Journal Requirements:**

*When submitting your revision, we need you to address these additional requirements.*

*1. Please ensure that your manuscript meets PLOS ONE's style requirements, including those for file naming. The PLOS ONE style templates can be found at*

[*https://journals.plos.org/plosone/s/file?id=wjVg/PLOSOne_formatting_sample_main_body.pdf*](https://journals.plos.org/plosone/s/file?id=wjVg/PLOSOne_formatting_sample_main_body.pdf) *and*

[*https://journals.plos.org/plosone/s/file?id=ba62/PLOSOne_formatting_sample_title_authors_affiliations.pdf*](https://eur01.safelinks.protection.outlook.com/?url=https%3A%2F%2Ftrack.editorialmanager.com%2FCL0%2Fhttps%3A%252F%252Fjournals.plos.org%252Fplosone%252Fs%252Ffile%253Fid%3Dba62%252FPLOSOne_formatting_sample_title_authors_affiliations.pdf%2F1%2F010f01953d65177f-9dfea311-5b4e-491c-84f3-79b5e120b825-000000%2FkuxSVrZRV0F7FgR2USZ7ko45NxsDO1idigBaiqeat5c%3D200&data=05%7C02%7C%7Cf01db20c9c1a44e8548108dd55a4358c%7C677181082eb7474ca1d741655d4b1e61%7C0%7C0%7C638760887600246763%7CUnknown%7CTWFpbGZsb3d8eyJFbXB0eU1hcGkiOnRydWUsIlYiOiIwLjAuMDAwMCIsIlAiOiJXaW4zMiIsIkFOIjoiTWFpbCIsIldUIjoyfQ%3D%3D%7C0%7C%7C%7C&sdata=t47Tcwj1f76um%2FKcx3DzKi5CJAMlQnEf7ZjtDTKsB1w%3D&reserved=0)

Response 1:

Thank you for your comment. I have revised **the entire manuscript** to ensure it meets PLOS ONE’s style requirements.

*2.* [*Thank you for stating in your Funding Statement:
“This research was partially supported by the internal research fund of Finnmark Hospital Trust. None of the funding providers had any role in the data analyses and interpretation, nor had they any right to approve or disapprove the writing and publication of the manuscript.”

Please provide an amended statement that declares *all* the funding or sources of support (whether external or internal to your organization) received during this study, as detailed online in our guide for authors at http://journals.plos.org/plosone/s/submit-now. Please also include the statement “There was no additional external funding received for this study.” in your updated Funding Statement.
Please include your amended Funding Statement within your cover letter. We will change the online submission form on your behalf.*](Thank%20you%20for%20stating%20in%20your%20Funding%20Statement:%20%0b)

Response 2:

Thank you for your feedback. I have updated the Funding Statement as follows **(page 33, lines 3-9)**:

“This research was supported by the internal research fund of Finnmark Hospital Trust. Additionally, the study was made possible through research time approved by my affiliated institution, the Sámi Norwegian National Advisory Unit for Mental Health and Substance Use (SANKS). None of the funding providers had any role in the data analyses and interpretation, nor had they any right to approve or disapprove the writing and publication of the manuscript. There was no additional external funding received for this study.”

*3. Thank you for stating the following in your Competing Interests section:
“The author declares no conflicts of interest.”

Please complete your Competing Interests on the online submission form to state any Competing Interests. If you have no competing interests, please state "The authors have declared that no competing interests exist.", as detailed online in our guide for authors at* [*http://journals.plos.org/plosone/s/submit-now*](http://journals.plos.org/plosone/s/submit-now) *This information should be included in your cover letter; we will change the online submission form on your behalf.*

Response 3:

Thank you for your comment. I have included the statement: “The author has declared that no competing interests exist” in both the ***Competing Interests*** **(page 33, lines 10-11)** and the cover letter**.**

*4. We note that you have indicated that there are restrictions to data sharing for this study. For studies involving human research participant data or other sensitive data, we encourage authors to share de-identified or anonymized data. However, when data cannot be publicly shared for ethical reasons, we allow authors to make their data sets available upon request. For information on unacceptable data access restrictions, please see* [*http://journals.plos.org/plosone/s/data-availability#loc-unacceptable-data-access-restrictions*](http://journals.plos.org/plosone/s/data-availability#loc-unacceptable-data-access-restrictions)*.

Before we proceed with your manuscript, please address the following prompts:

a) If there are ethical or legal restrictions on sharing a de-identified data set, please explain them in detail (e.g., data contain potentially identifying or sensitive patient information, data are owned by a third-party organization, etc.) and who has imposed them (e.g., a Research Ethics Committee or Institutional Review Board, etc.). Please also provide contact information for a data access committee, ethics committee, or other institutional body to which data requests may be sent.*

*b) If there are no restrictions, please upload the minimal anonymized data set necessary to replicate your study findings to a stable, public repository and provide us with the relevant URLs, DOIs, or accession numbers. Please see* [*http://www.bmj.com/content/340/bmj.c181.long*](http://www.bmj.com/content/340/bmj.c181.long) *for guidelines on how to de-identify and prepare clinical data for publication. For a list of recommended repositories, please see* [*https://journals.plos.org/plosone/s/recommended-repositories*](https://journals.plos.org/plosone/s/recommended-repositories)*. You also have the option of uploading the data as Supporting Information files, but we would recommend depositing data directly to a data repository if possible.

Please update your Data Availability statement in the submission form accordingly.*

Response 4:

Thank you for your feedback regarding the data availability statement. We have revised the statement to clarify the restrictions on sharing the data due to ethical and legal considerations. The dataset contains sensitive information and is subject to data protection regulations. Therefore, access is restricted and requires approval from the Norwegian Agency for Shared Services in Education and Research (Sikt) and Norwegian Social Research (NOVA).

For researchers interested in accessing the data, we have provided a link to Sikt’s Survey Bank, where data access requests can be submitted: <https://surveybanken.sikt.no/no/study/NSD3157/1?file=9c72d168-67ae-4c15-82d9-738535771af9/4&type=studyMetadata>.

**(see page 33, lines 12-21)**

**Comments from the Reviewer #1:**

*The manuscript "Assessment of the influential mechanisms of adolescent risk-taking behaviors and
protective and risk factors among high school students in Finnmark, Arctic Norway" by Shiho Hansen is about an important topic as there is only little or no reserach on substance use and anti-social behaviours among Sami adolescents. However, the manuscript has many shortcomings which need to be solved before it can be accepted for publication.

To begin with, there is a lot of research in the associations of various peer, family and school related risk and protective factors related to anti-social behaviours. It should be more clerly stated, what's new about this study, why would Sami adolescents be an interesting group to study? Do you suspect that this group would be somehat different or have different living conditions from those in the rest of Norway?

It is interesting that the trends of substance use among adolescents look similar in Finnmark as in rest of Norway, but there are differences with regard to trends in anti-social behaviours (asb). Thus, to me it looks like it would be more interesting to study asb and variables associated to it and try to reflect the results against other Norwegian adolescents. Would it be possioble to get the whole Ungdata data set and compare Sami adolescents with others?

A technical problem is that there are too much both outcomes and explanatory variables (92 in total!). Choosing only either substance use or asb (both include several separate measures as outcomes). would make the analysis simpler and the ms easier to follow. In addition, as substance use and other risk behaviours have often been shown to be interrelated, there is no need to take all these variables in the analysis. I also wonder, why you need so many explanatory variables. For example, what is the reason for including the "covid" variable? To continue, variables listed in Table 1 don't seem to be "latent variables". Did you use LCA or some other way of constructing latent variables? . See for example:
Lanza, S. T., & Rhoades, B. L. (2013). Latent class analysis: an alternative perspective on subgroup analysis in prevention and treatment. Prevention science : the official journal of the Society for Prevention Research, 14(2), 157–168.* [*https://doi.org/10.1007/s11121-011-0201-1*](https://doi.org/10.1007/s11121-011-0201-1)*.

To conclude, I suggest simplifying the manuscript, and comparing Sami adolescents against other adolescents in Norway. I also think more theoretical background is needed when rationalizing the research questions and hypothesis. In its present form the ms is more like a report of all that is included in Ungdata in a sample of Sami adolescents. If everything needs to be reported, I suggest writing 2-3 separate articles.*

**Response to Reviewer #1:**

Thank you for your insightful and constructive feedback. I have made the following revisions based on your suggestions. Changes occurred with refinement of outcomes and explanatory variables have been applied throughout the manuscript.

1. Refinement of Outcomes and Explanatory Variables:
   - The number of outcome and explanatory variables has been reduced to align with Bronfenbrenner's Social-Ecological Model, which underpins this study.
   - The focus is now on alcohol use (substance use) and rule-breaking behaviors (antisocial behaviors) due to their higher prevalence and significant health implications. The distinction between alcohol use and antisocial behaviors is discussed in the Discussion section.
2. Explanatory Variables:
   - Variables not central to the study, such as positive feelings and quality of life, have been removed due to their weak associations with the selected outcomes.
   - The COVID-19 variable has been retained given its relevance, as data collection occurred during the pandemic (2021).
3. Comparison with National Data:
   - A comparative analysis between adolescents in Finnmark and the national adolescent population is covered in a separate manuscript (*insert title*).
   - To maintain focus and avoid redundancy, this study does not include such comparisons.
4. Theoretical Background:
   - ***Background*** and ***Theoretical and Contextual Framework*** has been expanded to strengthen the rationale for the research questions **(page 2, line 19 – page 8, line 8).**

**Comments from the Reviewer #2:**

*The manuscript addresses a pertinent topic and takes a holistic approach to understanding the individual and environmental factors (family, school, peers) that may contribute to risky behaviors (substance use, antisocial behavior) among Norwegian adolescents from the Finnmark region.

The data from "Ungdata 2021" were used, allowing for a considerable sample size (2,129), making the results more robust. Sophisticated statistical analyses (PLS-SEM) with regression were used in the data analysis, allowing for exploring complex relationships between variables.

The results indicate that spending time with friends in the evenings is strongly associated with alcohol and tobacco use and rule violations. Family relationships influence rule violations and bullying behaviors, while school-related factors are only associated with rule violations. Neither family relationships nor school-related factors appear to be associated with substance use (i.e., alcohol, tobacco, or drug use).

However, as the authors also acknowledge, the study did not explore sociocultural and ethnic factors, which may play an important role in the population and context of the Finnmark region. The manuscript would benefit from providing more concrete data to support the relevance of this study in the specific region of Finnmark (e.g., data on delinquent behavior and substance use). In addition, more detailed information on that region's sociocultural characteristics could help contextualize or better discuss some of the results. Some results require further discussion or justification, especially those that contradict the research or are considered "surprising" (e.g., religiosity associated with alcohol consumption).

Overall, the manuscript addresses a relevant topic with potential implications for policy and practice in the specific region where the study was conducted. However, the authors must overcome some conceptual, methodological, and interpretative limitations for its publication. In this context, the manuscript requires major revisions.*

 *Important aspects to improve

- At the conceptual level, some constructs need to be clarified and better defined, namely "risk-taking behaviors," "protective factors," and "risk factors."*

Response 1:

Thank you for your valuable feedback. To improve conceptual clarity, we have explicitly defined *risk-taking behaviors*, *risk and* *protective factors* within the ***Model Development*** and ***Structure of the Model*** **(page 11, line 10 – page 15, line 14).** These definitions are now aligned with the theoretical framework of the study.

*- Explicitly acknowledge and discuss some limitations: the fact that this is a cross-sectional study does not allow for establishing causal relationships. For example, the study identifies correlations between interactions with peers and substance use; it is not possible to determine whether spending time with peers leads to increased alcohol consumption or whether young people who consume alcohol tend to spend more time with peers.*

Response 2:

Thank you for pointing this out. We have expanded the ***Limitation*** section to explicitly state that, while the findings indicate associations between peer interactions and substance use, the directionality of these relationships remains uncertain. We now clarify that causality cannot be inferred, as spending time with peers could lead to increased alcohol consumption, or vice versa **(page 31, lines 1-8).**

*- Several results contradict previous research or are paradoxical (e.g., religiosity positively associated with alcohol consumption, physical exercise positively associated with smoking). These results require more detailed explanations or justifications to avoid confusion. Review the statistical modeling process to ensure that these results are robust. If so, explanations based either on theory or on the specificities of the specific local context should be provided.*

Response 3:

Thank you for your observation. I appreciate your valuable feedback. To address this, I have taken several steps:

- Under ***Methods***, I have clarified the sample size determination to confirm the robustness of the analysis (***Partial Least Squares Structural Equation Modeling*** and ***Sample Size***, page 9 line 19 – page 10, line 25). In the ***Data Analysis*** section, I have explicitly discussed the reliability, validity, and multicollinearity assessments conducted prior to the PLS-SEM analysis **(page 15, line 14 – page 16, line 9)**. Additionally, I have included a description of bootstrapping with 10,000 resamples to ensure the robustness of parameter estimates **(page 16, lines 10-15)**.
- ***Discussion*** section has been revised **(page 16 line 8 – page 29, line 6)** to provide a more detailed analysis of the findings. This includes:
  1. Inconsistencies with previous research, such as religiosity not acting as a protective factor against alcohol use, a positive future outlook being linked to increased drinking, and no significant gender differences in alcohol use or antisocial behaviors;
  2. Negative associations observed between digital media use and risk behaviors, as well as strong peer relationships correlating with increased alcohol use;
  3. No associations found between family and school influences on alcohol use, individual factors and antisocial behaviors, socioeconomic status and risk behaviors, being bullied and risk behaviors, physical activity and risk behaviors, or attitudes toward antisocial behaviors.

*- Unlike substance use, attitudes related to antisocial behavior were not analyzed and are an important variable for understanding this type of behavior. It would be pertinent for the authors to consider the implications of this limitation when discussing some results.*

Response 4:

Thank you for pointing this out. We acknowledge this limitation and have explicitly stated in the ***Limitation*** section that attitudes toward antisocial behaviors were not assessed. We now discuss how this omission limits our ability to examine how individual beliefs and perceived social norms influence such behaviors **(page 31, lines 15-19).**

*- Several sections and many paragraphs do not present references to support some statements. For example, the section "Theoretical and Contextual Framework" only has one reference (about Bronfenbrenner's Social-Ecological Model), "Summary of studies in Norway"; the role of digital media in the formation of social norms. It is suggested to enrich the bibliographic review with relevant studies*

Response 5:

Thank you for your comment. I acknowledge the need to strengthen the support for statements. We have enriched the bibliographic review by incorporating additional references in key sections, including ***Summary of Studies in Norway* (page 5, line 15 -), *Theoretical and Contextual Framework* (page 6, line 19 -), and *Theoretical and Policy Implications* (page 29, line 7 -).**

*Other issues:

- Tables 2 and 3 present dense information that could be simplified, making them easier to read. The authors could consider highlighting key coefficients and removing non-significant paths to make them easier to read.*

Response 6:

Thank you for pointing this out. We have revised Tables 2 **(page 17, line 20 -)** and 3 **(page 20, line 16 -)** as follows:

- In Table 2, values related to loading, reliability, and validity are now highlighted in bold to emphasize their importance.
- In Table 3, significant associations are bolded, and negative significant associations are presented in bold and italics. Additionally, to simplify presentation, we have replaced p-values with asterisks (*) to indicate significance levels.

*- The presentation of p-values in tables 3a and 3b is not uniform. It is suggested that all significant p-values be presented with three decimal places to ensure uniformity.*

Response 7:

Thank you for bringing this to my attention. The R programming environment does not directly calculate p-values but instead provides t-values. The p-values were added based on the t-values for two-tailed tests. As mentioned in the previous comment, I have replaced the p-values with asterisks (*) to simplify the table’s formatting **(*Table 3 a-b,* page 20, line 16 -).**

*- In the "data analysis" section, the authors report, "This result suggests that PLS-SEM analysis can be applied across (78 out of 82 paths) of the path coefficients showed no statistically significant differences genders." It could enrich the work by discussing/explaining that there are no gender differences in the discussion or conclusion sections.*

Response 8:

Thank you for your comment. I completely agree and have added this point to **the *Discussion*** section under the ***Individual Factors*** subsection, highlighting the absence of gender differences (**page 25, line 22 – page 26, line 5)**.

**Additional Clarifications:**

**Minor English editing**: After revising the manuscript based on comments from the editor and the reviewers, **the entire manuscript** was proofread for clarity.

**Revision of Abstract**: Following the revision based on comments from the editor and the reviewers, I edited the **Abstract** to reflect these changes in the manuscript.
