# Supplementary material for: Assessment of the influential mechanisms of adolescent risk behaviors and protective and risk factors among high school students in Finnmark, Arctic Norway
Source: PLoS One. 2025 Jul 10;20(7):e0328066. doi: 10.1371/journal.pone.0328066 (PMC12244561; doi:10.1371/journal.pone.0328066)
Supplement: Attachment — Submitted filename: Response to the Reviewer Comments 2.docx [file pone.0328066.s003.docx]

**Response to the Reviewers’ Comments and Concerns**

*Reviewer #1: The manuscript "Assessment of the influential mechanisms of adolescent risk behaviors and protective and risk factors among high school students in Finnmark, Arctic Norway" has been improved a lot since my late previous review. The theoretical background has been added and it works well. Still, part of my comments or suggestions have not been considered, most importantly the definition of "latent variables". I still feel that this must be taken into account to avoid confusion as here the term "latent variable" has not been used in a standard way as in latent class analysis. In this round I also go more into details.

In its present form the structure of the manuscript is somewhat disordered. Although the journal doesn't have any explicit requirements for section organization, I suggest ordering the manuscript as Follows:
Introduction

Introduction
Background
Alcohol use among Norwegian adolescents (instead of "Alcohol use in Norway")
Antisocial behavior among Norwegian adolescents (instead of "Antisocial behaviour in Norway)
Risk and protective factors for alcohol use and antisocial behavior (instead of "Summary of studies in Norway")
Finnmark’s Unique Context and Adolescent Risk Behaviors
Theoretical and Contextual Framework
Purpose of the Study

- I think the section "Summary of studies in Norway" can be removed as it mostly repeat what is already said in the two previous sections.

Data and methods (instead of Methods)
Data (this includes "Data Source and Samples", "Sample Size" and "Measures")
Statistical methods (this includes "Partial Least Squares Structural Equation Modeling", "Model Development", "Structure
of the Model" and "Data analysis"
Ethics

Results
Evaluation of Measurement Models
Alcohol use
Antisocial behavior
(these two instead of "Evaluation of Structural Models")

Discussion
Environmental Factors
Individual Factors
Adverse Experiences
Leisure time activities (instead of Digital Media Use and Physical Activities as Free Time Use)
Theoretical Implications
Political Implications
Strengths and Limitations (probably there are also strengths in this study?)
Conclusion

- I suggest removing the section "Attitudes and Behaviors" as they are not really measured here.*

**Response:**

Thank you for your helpful suggestions regarding the manuscript structure. In response, I have reordered and renamed the relevant subsection titles according to your proposed outline. I have also removed the sections “Summary of Studies in Norway” as it was repetitive or not directly relevant to the core focus of the study. The section “Attitudes and Behaviors” was also removed—not only based on your suggestions, but also due to the exclusion of variables related to attitudes toward alcohol use (see the detailed response to comments concerning individual variables and their naming).

***Specific comments on the Introduction:***
 *p3, l12-15: "Understanding the interplay between individual factors, environmental influences (family, school, peers), and regional contexts—as seen in Finnmark, Norway—can provide insights into how digital and social media shape adolescent health outcomes." - This sentence feels a bit loose as it is not the focus of this study and no answers to this are offered. I suggest removing it.

p8, l10-15: The purpose of the study should be rewritten avoiding terms like "underlying mechanisms" and "influence". As this study uses cross-sectional data, causality can't be studied. So, I suggest using terms like associations or relationships or otherwise avoid referring to causality.

p9, l13-14: The grades may not open up to other than Norwegian readers, present also ages of students.*

**Response:**

Thank you for your valuable comments on the Introduction.

p. 3, l. 12–15 (Version 1): I have removed the sentence as suggested. It was located just before the subheading “Alcohol Use Among Norwegian Adolescents” (p. 3, l. 39).

p. 8, l. 10–15 (Version 1): I have revised the purpose statement to avoid terms such as “underlying mechanisms” and “influence.” Instead, I have used terms like “associations” and “relationships” (see *Purpose of the Study*, p. 7, l. 22–p. 8, l. 3).

p. 9, l. 13–14 (Version 1): While the Ungdata survey did not include students’ exact ages, I have now indicated the typical age range corresponding to each school grade to clarify this information for an international audience (*Data Source and Samples*, p. 9, l. 8–12).

***Specific comments on Data*** *The description of all variables should be presented under the "Measures" section. Now they are under "Model development" and "Structure of the Model". In section "Data source and samples", also class and gender are mentioned. As all readers may not be familiar with the Norwegian school system, I suggest presenting age together with class.*

**Response**

Thank you for the insightful and constructive feedback.

**Placement of Variable Descriptions (Measures section)**

As responded in the previous comments, I have reordered and renamed the relevant subsection titles including “*Measures*” section (s.9, l.25 – s.14, l.1).

I agree that all variable descriptions should be consolidated under a dedicated "Measures" section for clarity and consistency. In the revised manuscript, the full description of measured and composite variables has been relocated to a newly organized “*Measures*” section, which now includes all relevant details previously presented across multiple sections (“Model Development” and “Structure of the Model”).

**Clarification of School Grade and Age**
As responded in the previous section, while the Ungdata survey did not include students’ exact ages, I have now indicated the typical age range that corresponds to each school grade to make the information clearer to an international audience (*Data Source and Samples*, p. 9, l. 8–12).

*- The construction of latent variables is not clear. It looks like the individual variables that have been included in the latent variables have been selected by the author and not by using for example latent class analysis (LCA) as suggested by Lanza (e.g. Lanza, S. T., & Rhoades, B. L. (2013). Latent class analysis: an alternative perspective on subgroup analysis in prevention and treatment. Prevention science: the official journal of the Society for Prevention Research, 14(2), 157–168.*
[*https://doi.org/10.1007/s11121-011-0201-1*](https://doi.org/10.1007/s11121-011-0201-1)*). I suggest finding some other term than "latent" for variables that have been constructed from several variables. In addition part of the "latent" variables are same as individual variables (e.g. SES, religion) and thus can't be considered as latent.

It is also unclear, how the "latent" variables were constructed in practice. For example, on p14 (l10-14) it is stated that "BALKO refers to risky behaviors related to alcohol use and comprises five measured variables, including having consumed any type of alcoholic beverage, having consumed enough alcohol to feel clearly intoxicated, and having driven after drinking alcohol. These behaviors are measured by the variables “balko1” to “balko5." - How is this "latent" variable then created? A sum of all the listed variables? And how it is decided that these variables together form a "latent" variable? The same goes for all the "latent" variables. Please open this up!*

**Response**

I appreciate the suggestion regarding terminology.

**Use of the Term “Latent Variable”**
To avoid confusion with statistical latent variable models (e.g., those derived from latent class analysis), we have replaced the term *latent variable* with ***composite variable*** throughout the manuscript. This term better reflects the construction process used in our study, which was theory-driven and based on the thematic grouping of items from the Ungdata survey, not data-driven statistical clustering methods like LCA.

**Construction of Composite Variables**
The construction of each composite variable has been explicitly described in the revised "Measures" section. For example, the composite variable “alcohol use” consists of three observed indicators. Each item was standardized, and the composite score was computed using the PLS-SEM algorithm, which calculates weighted linear combinations of indicators based on their outer loadings. The rationale for grouping these items was informed by both theoretical constructs and prior empirical applications within the Ungdata framework. These construction steps have been detailed (*“Measures”* p. 10, l.12-18).

*- The description of individual variables is not sufficient either, as the response options are not offered. I understand that it is not possible to write out all the used 65 variables (not 92 anymore, p11, l3), but could you offer a reference to the Ungdata questionnaire or if this is not possible, describe the variables (original questions with response options) as a supplement? The description of how socioeconomic status was measured is missing altogether. Finally, it is not necessary to present the variable names (individual and latent) in Table 1.*

**Response**

**Measurement of Socioeconomic Status (SES)**
I included a detailed description of how socioeconomic status (SES) was measured. In this study, SES was assessed using an index developed specifically for the Ungdata survey that comprises three key dimensions: parental education, the number of books in the home, and indicators of family affluence (*“Measures”* p.10, l.19-p.11, l.2).

**Description of Individual Variables and Response Options**
Given the extensive number of variables used in the analysis, we recognize the impracticality of listing all question wordings and response categories in the main text. In response, Table 1 has been revised to include the original item wordings and corresponding response options (translated into English) for all measured and composite variables used in the model. This approach replaces the earlier version of Table 1, avoiding making a separate supplementary table while ensuring transparency and clarity. Although the complete Ungdata questionnaire cannot be reproduced due to data access restrictions, all items relevant to this study are now presented in Table 1 with appropriate attribution (p.12, l.12 – p.14).

*Finally, I wonder if age was standardized in the analysis? If not, it should at least be discussed as the age range seems to be at least three years (the data includes three class levels). In adolescence, three years (or more?) makes a big difference with regard to the studied outcomes.*

**Response**

I acknowledge the concern about the potential confounding influence of age, particularly given the developmental variability across adolescence. Although age was not standardized in the PLS-SEM model due to the absence of direct age data in the Ungdata survey, we have now addressed this limitation explicitly in the “Limitation (p.32, l.23 – p.33, l.4)”. As a proxy, school grade was used, but I recognize that intra-grade age variation may exist and could influence certain outcomes. This limitation has been noted, and recommendations for future data collection practices are offered.

***Specific Comments on Variable Naming and Content***

*The following comments are related to individual variables or their names.
1) "Depression": I would call this construct "depressive symptoms" rather than "depression", which refers to a diagnosis (that is not measured here).
2) "Digital use": In table 1 this is described as "Playing computer games/video games". If this is what the really measures, a better name for this variable would be "gaming". However, on p27, l8-9 digital media use is defined as "online gaming and video watching". So, which one is correct? If also video watching is included, I think it is somewhat questionable to put these two into one question or variable as they are qualitatively quite different. Videos are also watched via social media platforms so this can partly be confused with social media use.
3) "Trust between parents": The name of this variable sounds like it measures trust between parents and not how parents trust their children. In research literature, this is often called e.g. parental monitoring and I suggest using this term also here.
4) "School": I think a more correct name for this would be e.g. "Liking school".
5) "Attitude toward drinking": I think this is particularly problematic, as it really doesn't measure attitude toward drinking but toward drunk driving. In going through the results and discussion, this is however considered as presenting attitude toward alcohol use. This should be corrected throughout the manuscript and also mentioned as a limitation when discussing attitudes (p28, l7-13).
6) "Smoking": as this includes also snus and e-cigarette, I suggest naming this as "Tobacco and nicotine product use" as is the common habit in the research literature.
7) "Drinking": I don't think being a passenger of a drunk driver should be included here as it doesn't measure own alcohol use. I would also consider leaving drunk driving out of this "latent" variable. At least you should test whether the results of the main analysis on alcohol use remain the same if these two are excluded, and if you decide to include them, present some reasoning for this decision. To continue, I'd call this measure "alcohol use" rather than "drinking".
8) "Drug use": As with alcohol use, I don't think being offered cannabis measures drug use. Also here you should test how removing this item affects the results and present reasoning for including this variable if you end up in doing so.
9) "Aggressive behavior": Why is selling drugs included here? Again, test the "latent" variable without this item and explain the inclusion criteria.*

**Response**

We sincerely appreciate your careful review and thoughtful suggestions regarding the naming and content validity of individual variables. Below are our point-by-point responses and corresponding revisions:

1. **"Depression"**
   The term *“depression”* has been revised throughout the manuscript to *“depressive symptoms”*, as we agree this more accurately reflects the nature of the construct measured, which does not represent a clinical diagnosis.
2. **"Digital use"**
   We acknowledge that our earlier description was imprecise. The Ungdata questionnaire includes two distinct items: (1) *“How much time do you spend playing computer games/video games?”*—which we now clearly refer to as **gaming**, and (2) *“How much time do you spend on social media (Facebook, Instagram, etc.)?”*—which we refer to as **social media use**. The previously mentioned inclusion of “video watching” was incorrect and has been removed to avoid confusion, as this activity is not separately measured in the questionnaire and could overlap with social media use. I have revised the terminology throughout the manuscript to ensure consistent and accurate use of these labels and have addressed this limitation in the discussion section.
3. **"Trust between parents"**
   The label *“trust between parents”* has been revised to *“parental monitoring”* throughout the manuscript, which more accurately reflects the construct, referring to parental awareness and supervision of their child’s activities, consistent with the literature.
4. **"School"**
   This variable was labeled *“liking school”* throughout the manuscript, which more precisely captures the intent of the items used and aligns with previous studies on school engagement and well-being.
5. **"Smoking"**
   This variable has been updated to *“tobacco and nicotine product use”* throughout the manuscript, which encompasses traditional smoking, snus, and e-cigarettes, aligning with contemporary terminology in public health research.
6. **“Attitude Toward Drinking (or Attitudes Toward Drunk Driving),” “Drinking (or Alcohol Use),” and “Aggressive Behavior”**

**6.1. Rewording of Constructs**
The construct previously labeled *“Drinking”* has been renamed *“Alcohol Use”* throughout the manuscript to more accurately reflect the behavior measured.

*“Attitude Toward Drinking”* was ultimately removed due to both a conceptual mismatch and the lack of a significant association with alcohol use in the revised model (see Section 6.4 below for further details).

**6.2. Exclusion of Specific Items and Reanalysis**
In response to the reviewer’s comments, the following items were excluded from their respective composite variables, and the PLS-SEM analyses were re-run (see Table 1, p.12, l.12 – p.14):

- *“Being a passenger of a drunk driver”* and *“Drunk driving (as a driver)”* were removed from the **Alcohol Use** composite.
- *“Being offered cannabis”* was excluded from the **Drug Use** composite.
- *“Selling drugs”* was removed from the **Aggressive Behavior** composite.

**6.3. Effects on Model Outcomes**
Following these modifications, the revised analysis revealed that two previously significant associations were no longer statistically significant:

- The association between *Attitude Toward Drunk Driving* and *Alcohol Use*.
- The association between *Drug Use* and *Antisocial Behavior (Table 3 b, p.22-23)*.

**6.4. Removal of “Attitude Toward Drunk Driving” from the Model**
Given both the conceptual misalignment and the absence of a significant relationship with alcohol use, the construct *“Attitude Toward Drunk Driving”* was removed from the model. A final round of PLS-SEM analysis confirmed that the overall model results remained consistent with those observed in the earlier revised analyses (Sections 6.2 and 6.3).

***Specific comments on Results***

*In Tables 2, 3a and 3b, present what is measured instead of variable names. To help reading the tables, the same subheadings could be provided in tables as in Discussion (Environmental Factors, Individual Factors, Adverse Experiences, Leisure time activities) and the variables grouped under them. The same order could be used in writing out the results.*

**Response:**
I have revised Tables 2 (p.19-20) , 3a, and 3b (p.22-23) by replacing variable names with the corresponding measured constructs (e.g., "Religiousness" instead of "RELIG"). Subheadings have been added to align with those used in the Methods and Discussion sections. Variables are now grouped under these subheadings, and the order of composite variables has been standardized across the Methods, Results, and Discussion sections to improve clarity and consistency. The unified order is: Psychosocial Background, Adverse Experiences, Leisure Time Activities, Environmental Factors, and Other Risk Behaviors.

*Partly related to the description of variables - the presentation of results need clarification. The direction of the associations is mostly not clear as you use expressions like "The final model showed positive associations with religion (RELIG; β = 0.114), depression (DEPR; β = 0.053)...” For example, did religiousness increase or decrease the risk of alcohol use? Please go through all the results paying attention to this.*

*I would avoid using variable names in reporting results, but instead use expressions related to what was measured. So, this sentence could be easier to read if reformulated as follows: "A high level of religiousness increased the risk of alcohol use (β = 0.114). Also depression (or depressive symptoms?) increased the risk (β = 0.053)..."*

**Response**
Thank you for the feedback. I have revised the Results (p.21, l.2 – p.22, l.6) to clearly state the direction of each association (e.g., increased or decreased risk) and replaced variable names with descriptive labels for the measured constructs (e.g., “religiousness” instead of “RELIG”).

***Specific comments on Discussion***
*p22, l12-13: "Interestingly, peer relationships showed a negative association, suggesting that greater trust in friends correlates with increased alcohol use." - Only one question in the VENN variable is about trust, it seems that this construct rather measures having friends and not trust. Please correct this.*

**Response:**
Thank you for the clarification. The construct labeled "VENN" primarily reflects the presence and availability of friends rather than trust. Accordingly, I revised the description to refer to *friendship support* instead of *trust* throughout the manuscript to more accurately represent what was measured in this study.

*p22, l21: "...descriptive norms of drinking (peers' actual drinking behaviors) have consistently been found to predict adolescent alcohol use. Moreover, descriptive norms significantly mediated the relationship between drinking with peers and alcohol use for both males and females, with a somewhat larger effect observed among males."
- This is somewhat complicated language, maybe just say that peers' drinking behavior is associated with own alcohol use?
p23, l1-2: "injunctive norms (perceived approval of drinking) shaped both immediate and future drinking behavior, highlighting the role of social conformity."
- Again really complicated language, simply perceived (peers?) approval of drinking?*

**Response**
Thank you for the helpful suggestions. The terms "descriptive norms" and "injunctive norms" have been simplified to "peers' drinking behavior" and "perceived peer approval of drinking," respectively, to better convey their meaning and relevance to adolescent alcohol use (p.28, l.18-25).

*p23, l10-11: "These regional differences highlight the varying impact of family and school environments on adolescent behavior across Norway."
- Where the used measures similar? It appears that no. I would be careful in making conclusions like this with different data and measures.*

**Response**

Thank you for this important observation. I agree that differences in measures and study designs across regional studies limit the strength of direct comparisons. In response, we revised the sentence to adopt a more cautious tone (p.29, l.8-10).

*p24, l1-4: "This dynamic may help explain why spending evenings with friends is strongly associated with multiple risk behaviors in Finnmark. Increased unsupervised time during the evenings may heighten exposure to delinquent peers, thereby increasing the likelihood of engaging in risky behaviors [44], including alcohol use and antisocial activities".
- Maybe because of less possibilities for organized activities than in e.g. the metropolitan area?*

**Response**

Thank you for your comment. Upon review, I found that the paragraph in question repeated points already addressed earlier regarding unstructured peer interaction and associated risk behaviors. To improve clarity and avoid redundancy, I have removed it from the manuscript (p.28, l.1-10).

Regarding your suggestion about organized activities, I have incorporated relevant findings from a national trend analysis showing that adolescents in Finnmark participate less frequently in organized leisure activities than the national average. This information has been included in the first paragraph of the section, where it is more thematically appropriate (p.28, l.1-10).

*p24 (Individual factors): In this chapter there is again a lot of unclear expressions related to the direction of associations. For example "...future outlook showed a negative association with alcohol use". Future outlook -> positive future outlook. This is actually said in other words on p25, l12-13 (repetition), but the expression is more clear here.*

*p27 (Leisure activities): Again, rewrite this section so that the direction of associations becomes clear.*

**Response**

I have revised both sections to clarify the direction of associations and ensured consistency in phrasing (p.24, l.1-5, p.26, 15-19).

*p26, l7-8: "...bullying others was associated only with antisocial behaviors".
- Isn't bullying others a type of anti-social behavior? In addition, I don't think it should be considered an "adverse experience" as other experiences under this title refer to something that one has been exposed to and not something that one has been doing him-/herself. Please consider, whether this need to be studied as a separate variable or could it be combined under the outcome "antisocial behaviors.*

**Response**

Thank you for your feedback. Based on your suggestion, I have moved bullying under "Other Risk Behaviors" instead of categorizing it as an "adverse experience" (Table 1, p.12, l.12 – p.14). While bullying others can indeed be considered a form of antisocial behavior, I have distinguished it as a separate variable in this study. The antisocial behaviors we focus on are specifically rule-breaking actions, such as stealing, not paying for services (e.g., cinema tickets, bus fare), skipping school, or cheating on tests, which align with the behaviors typically captured in the Ungdata survey. Bullying, on the other hand, was categorized separately because it relates to moral and ethical violations (e.g., causing harm to others) and was not included as a form of rule-breaking behavior in the Ungdata dataset. Therefore, I have kept bullying as a separate variable.

*p27, l1-2: "Finnmark’s isolation and limited activities [48] may explain why alcohol remains a coping mechanism."
- This is quite speculative. This study doesn't offer any evidence of alcohol as a coping mechanism.*

**Response**

Thank you for your feedback. This study does not provide direct evidence of alcohol as a coping mechanism. I removed this sentence to avoid making unsupported claims (paragraph, p.25, l.3-10).

*p27, l12-13: "Since spending time with friends in the evening was associated with increased alcohol use and antisocial behaviors [7, 18], reduced in-person socializing due to digital engagement may explain the inverse relationship."
- But are these negatively associated? So that those who spend a lot of time with peers do not spend that much time on digital activities? Did you test this? What if they watch videos together with friends? I think this reasoning is quite weak and should be rewritten.*
**Response**

Thank you for your comment. I have revised the paragraph to clarify the reasoning and included evidence from a recent Ungdata-based study, which found that more time spent with friends in the evening was associated with less time spent gaming. This supports the interpretation of a potential displacement effect, while acknowledging that shared digital activities may also occur (p.25, l.24 – p.26, l.4).

*p27, l18-19. "36, 37]. "Since social media amplifies peer influences, adolescents may adopt drinking behaviors as a form of social integration."
- Please provide scientific evidence with references for this line of reasoning. It is also argued that time spent in social media is away from time spent with friends irl. As drinking among adolescents mostly is a social activity, more time spent in social media instead of irl would decrease alcohol use.*
**Response**

Thank you for your comment. I have removed the sentence to avoid speculative reasoning, as the claim cannot be directly supported with scientific references (p.27, l.5-15).

*p27, l24-25: "Regional differences, such as those in Finnmark, where social opportunities are more limited [48], may also contribute to these findings."
- What differences? Maybe regional characteristics? And how would they contribute to your findings. Please open up.*
**Response**

Thank you for your comment. I agree that the original phrasing could be more specific. I will revise the sentence to clarify that I am referring to regional characteristics in Finnmark, such as geographic isolation and a sparse population (p.25, l.3-10).

*p28 (Attitudes and behavior) Attitudes toward drunk driving is not the same thing as attitudes toward drinking and referring to studies on perceived access of alcohol are really out of context here. The lack of measuring attitudes should rather be discussed in limitations.*
**Response**

As mentioned in the **Response 6.4 “Removal of “Attitude Toward Drunk Driving” from the Model**,” *“Attitude Toward Drunk Driving”* was removed from the model given the conceptual misalignment.

*p29, l16-18: "The study also supports the theory of planned behavior [59] by showing that attitudes toward alcohol use predict drinking behaviors..."
- As you didn't actually measure this attitudes toward alcohol use but toward drunk driving, I don't think you can say this. In addition, no causal inferences can be made with this cross-sectional data (attitudes predict drinking).*

**Response**

As mentioned in the **Response 6.4 “Removal of “Attitude Toward Drunk Driving” from the Model**,” *“Attitude Toward Drunk Driving”* was removed from the model given the conceptual misalignment. Relevant description was therefore removed from the Discussion.

*p30, l8: "Recognizing the association between digital media and alcohol use..."
- Social media, not digital media? However, as there is evidence of both negative and positive associations between social media use and alcohol use, this section should be elaborated.*
**Response**

Thank you for your comment. I have revised the section to specify social media rather than digital media and elaborated on the contrasting associations (p.31, 11-16)—highlighting that while social media use was linked to increased alcohol use, gaming showed a negative association, possibly reflecting reduced offline socializing where risk behaviors often occur.

*p31, l8: "Attitudes toward alcohol use were identified as a key factor in shaping drinking behaviors, consistent with the theory of planned behavior."
- Again, you didn't measure attitudes toward alcohol use but drunk driving! And I don't see how this is consistent with the theory of planned behavior, as mentioned earlier, causality can't be inferred here. Please remove this conclusion.*
**Response**

As mentioned in the **Response 6.4 “Removal of “Attitude Toward Drunk Driving” from the Model**,” *“Attitude Toward Drunk Driving”* was removed from the model given the conceptual misalignment. Relevant description was therefore removed from the Conclusion.

*p31, l12-13: "Cultural factors, such as Sámi parenting practices emphasizing autonomy, may also contribute to the unique behavioral patterns observed in the region."
- I would be careful in these kinds of interpretations. Looking at Ungdata results, parenting related variables don't seem to be very different in Finnmark compared to other parts of Norway, rather there seems to be variation between communities all around the country, also in Finnmark.*

**Response**

Thank you for your feedback. Based on your suggestion, I removed the sentence to avoid making unsupported interpretations (p.31, l8-11).

**Additional Clarifications:**

**Minor English editing**: After revising the manuscript based on comments from the editor and the reviewers, **the entire manuscript** was proofread for clarity.

**Revision of Abstract**: Following the revision based on comments from the reviewer, I edited the **Abstract** to reflect these changes in the manuscript.
